# Prognostic value of stress cardiovascular magnetic resonance in patients with ischaemic heart disease and severely reduced left ventricular ejection fraction

**DOI:** 10.1136/openhrt-2025-003466

**Published:** 2025-08-26

**Authors:** Ailís Ceara Haney, Janek Salatzki, Andreas Ochs, Thomas Hilbel, Lukas D Weberling, Hauke Hund, Evangelos Giannitsis, Norbert Frey, Henning Steen, Dirk Lossnitzer, Florian André

**Affiliations:** 1 Department of Cardiology, Angiology and Pneumology, Heidelberg University Hospital, Heidelberg, Germany; 2 German Centre for Cardiovascular Research, Heidelberg, Germany

**Keywords:** CORONARY ARTERY DISEASE, Cardiac Imaging Techniques, Magnetic Resonance Imaging, HEART FAILURE

## Abstract

**Background:**

The concept of ischaemia for therapeutic guidance and risk stratification in coronary artery disease has been challenged in recent years. In particular, there is limited understanding of the prognostic value of ischaemia in patients with severely reduced left ventricular ejection fraction (LVEF). The aim of this study was to investigate the prognostic value of stress cardiovascular magnetic resonance (stress CMR) in patients with ischaemic heart disease (IHD) and severely reduced LVEF.

**Methods:**

This retrospective study included patients with IHD and an LVEF ≤35% who underwent stress CMR between 2009 and 2022. The primary endpoint was the occurrence of a major adverse cardiovascular event (MACE), including cardiac death, non-fatal myocardial infarction (MI), survived sudden cardiac death and implanted cardioverter defibrillator shock for ventricular fibrillation. The secondary combined endpoint included heart failure hospitalisation, percutaneous coronary intervention, arrhythmia and coronary artery bypass grafting (CABG). All-cause death was also documented.

**Results:**

The study population consisted of 362 patients (85.6% men, 70.5 (63.0–78.0) years) with an LVEF of 30.2% (25.2%–33.0%). 245 patients (67.6%) had three vessel disease, 206 patients (57.2%) had a history of MI and 83 patients (22.9%) had a history of CABG. Stress CMR showed ischaemia in 72 (19.9%) patients. Among those, 32 patients (8.8%) underwent early revascularisation. Follow-up was 4.5 (3.0–6.6) years. MACE occurred in 101 patients (27.9%), including 41 cases of cardiac death (11.3%) and 40 cases of MI (11.0%). Ischaemia was not significantly associated with MACE, the combined secondary endpoint, or all-cause death in survival analysis (HR for MACE 1.20, 95% CI 0.74 to 1.95, p=0.4).

**Conclusion:**

In a cohort of patients with IHD and severely reduced LVEF, outcome did not differ when stratifying by ischaemia on stress CMR. We found no evidence that ischaemia could identify patients with increased risk for MACE, the combined secondary endpoint or all-cause death.

**WHAT IS ALREADY KNOWN ON THIS TOPIC**

- Ischaemia detected during cardiovascular magnetic resonance (CMR) in patients with known or suspected coronary artery disease (CAD) is associated with a worse prognosis. However, the prognostic significance of ischaemia in patients with ischaemic heart disease (IHD) and severely impaired left ventricular function remains poorly understood.

**WHAT THIS STUDY ADDS**

- In this retrospective study based on patients with left ventricular ejection fraction (LVEF) ≤35% due to extensive CAD who underwent CMR stress testing, we observed that evidence of ischaemia was not associated with cardiovascular death, non-fatal myocardial infarction or survived sudden cardiac death, among others. Less than half of the patients with ischaemia during stress testing received revascularisation within 3 months. More than one quarter of the population experienced a major adverse cardiovascular event during follow-up.

**HOW THIS STUDY MIGHT AFFECT RESEARCH, PRACTICE OR POLICY**

- In patients with IHD and severely reduced LVEF, management decisions, such as whether to pursue revascularisation, should go beyond assessing ischaemic burden alone, incorporating patient-specific factors, volumetric parameters and a shared decision-making process.

## Introduction

 Ischaemic heart disease (IHD) contributes significantly to the global burden of disease, accounting for significant premature mortality and substantial economic implications. The concept of ischaemia to guide revascularisation in chronic coronary syndrome and essentially to reverse hibernation, restore cardiac function and prevent myocardial infarction (MI) has been challenged by several large trials in recent years.^1^,^3^

Stress testing with cardiovascular magnetic resonance (stress CMR) demonstrated high sensitivity and specificity for detecting functional relevance of coronary artery disease (CAD) compared with other imaging modalities and is non-inferior to invasive fractional flow reserve measurements.^4^,^7^ Ischaemia detected during CMR in patients with known or suspected CAD is associated with a worse prognosis, including cardiac death and non-fatal MI.^8 9^

Yet, there is limited understanding of the prognostic value of ischaemia in patients with severely reduced left ventricular (LV) function, particularly in those with IHD. Previous studies assessed vasodilator stress CMR in patients with reduced left ventricular ejection fraction (LVEF), finding strong prognostic value for the occurrence of major adverse cardiovascular events (MACEs) with inducible ischaemia.^10 11^ However, most patients in these studies had only mildly or moderately reduced LVEF, with the underlying aetiology being both ischaemic and non-ischaemic.

A subanalysis of the STICH trial found that inducible ischaemia had no significant prognostic value in patients with LVEF ≤35% and CAD suitable for coronary artery bypass grafting (CABG). However, since stress testing was performed using exercise or dobutamine stress echocardiography as well as nuclear imaging, quantification of ischaemic burden differed between modalities.^12^

Therefore, the aim of this study was to assess the prognostic value of stress CMR in patients with severely reduced LV function due to ischaemic aetiology.

## Methods

### Study population

Patients with severely reduced LV function (LVEF ≤35%) assessed by CMR who underwent clinically indicated stress CMR at our centre between 2009 and 2022 were retrospectively enrolled in this single-centre study. Inclusion criteria were (1) presence of epicardial coronary vessels with ≥50% stenosis assessed by clinically indicated invasive coronary angiography (ICA) performed before or after stress CMR and any history of MI or coronary revascularisation (either percutaneous transluminal coronary angioplasty (PCI) or CABG) in patients with single-vessel disease, (2) completed clinically indicated stress CMR, including attaining 85% of age-predicted maximal heart rate during dobutamine/atropine stress CMR and (3) minimum follow-up of 2.5 years. Patients with cardiac amyloidosis were excluded due to their poor prognosis. Other forms of non-ischaemic cardiomyopathy, such as dilatative cardiomyopathy, were not reasons for exclusion when present alongside IHD. Of note, IHD had to be the primary cause of LV dysfunction.

In approximately half of the study population, laboratory results from the day of the exam ±7 days were available.

### CMR protocol

#### CMR image analysis

CMR was performed in a clinical 1.5 T (Achieva or Ingenia CX) or 3 T (Ingenia) whole body scanner (Philips Healthcare, Best, The Netherlands). Vasodilator stress CMR was performed using adenosine or regadenoson. An inducible ischaemia was visually deemed present in case of hypoperfusion of at least one segment according to the 16-segment model of the American Heart Association (AHA) during perfusion, not evident at baseline, which was accompanied by a perfusion/late gadolinium enhancement (LGE) mismatch in the presence of LGE.^13^

In cases where the accuracy of vasodilator stress may be reduced due to extensive scar tissue, balanced ischaemia, or uneven perfusion, eg,chronic total occlusion, dobutamine stress CMR was performed.^14^ Previous literature has demonstrated that dobutamine stress CMR and perfusion imaging offer comparable diagnostic accuracy.^15^ Prior work has particularly demonstrated the high sensitivity and specificity of the dobutamine stress test in patients with pre-existing wall motion abnormalities (WMAs) due to a prior MI and/or coronary artery revascularisation.^16^ Further reasons to perform dobutamine stress CMR were adenosine hypersensitivity or advanced chronic kidney disease defined as an estimated glomerular filtration rate of <15 mL/min/1.73 m² or haemodialysis.^17^ Inducible ischaemia was defined as new or worsening WMAs in at least one myocardial segment, evident in two views during stress.

First pass perfusion imaging during peak stress was performed in three LV short-axis slices (apical, mid-ventricular and basal) in selected cases. Inducible ischaemia was defined as visible hypoperfusion of at least 1 AHA segment during perfusion that was not present at baseline and in the absence of LGE in the same segment. When both inducible WMA and hypoperfusion were present, the number of segments was summed, provided they were located in different segments. If WMA and hypoperfusion occurred in the same segment, this was counted as one segment only.

Details on CMR imaging analysis and stress CMR are described in the online supplemental materials.

### Coronary angiography

Patients with known or suspected obstructive CAD underwent clinically indicated ICA either prior to stress CMR and were referred for stress CMR due to moderate to severe stenosis of unknown haemodynamic relevance or after stress CMR because of ischaemia detected during stress testing or due to persistent symptoms despite negative stress test results. The examinations were visually analysed by two experienced readers.

### Patient follow-up and outcome

Follow-up was carried out through electronic medical records or direct contact with the patient’s primary care physician or the patient. The primary endpoint was the occurrence of a MACE, defined as cardiac death, survived sudden cardiac death, adequate shock by implantable cardioverter defibrillator (ICD) due to ventricular fibrillation, or non-fatal MI. The combined secondary, non-hierarchical, endpoint included hospitalisation for heart failure (HF), PCI, CABG or non-fatal arrhythmia defined as ventricular tachycardia (VT) requiring electrical cardioversion or adequate ICD-shock for VT. Furthermore, all-cause death was assessed. Details on patient follow-up and outcome are described in the online supplemental materials.

### Patient and public involvement

Patients/the public were not involved in the research.

### Statistical analysis

Continuous variables were tested for normal distribution using the Shapiro-Wilk test and reported as mean±SD for normally distributed variables or median and IQR for non-normally distributed variables. Group differences were assessed using Student’s t-test or Mann-Whitney U test, as applicable. Categorical variables were tested using the χ^2^ test. Kaplan-Meier curves were used for visual representation of survival estimation as a function of follow-up time. Differences between groups were assessed using log-rank testing. An interim analysis was performed for MACE and for all-cause death after 1000 days, restricting the follow-up to 1000 days and excluding all events after 1000 days. Univariate HRs for endpoints were calculated using Cox proportional regression models. The primary survival analysis included all patients with ischaemia in the ischaemia group, regardless of whether they underwent early revascularisation. To minimise the potential effect of (early) revascularisation on subsequent clinical endpoints, an additional analysis was performed, reassigning patients who received revascularisation due to ischaemia to the no ischaemia group. Additionally, a Cox regression analysis was performed, censoring all patients undergoing early revascularisation. A p value of <0.05 was regarded as statistically significant. Statistical analysis was performed with MedCalc (V.20.114 MedCalc Software, Ostend, Belgium).

## Results

### Patient characteristics

**Figure 1:**
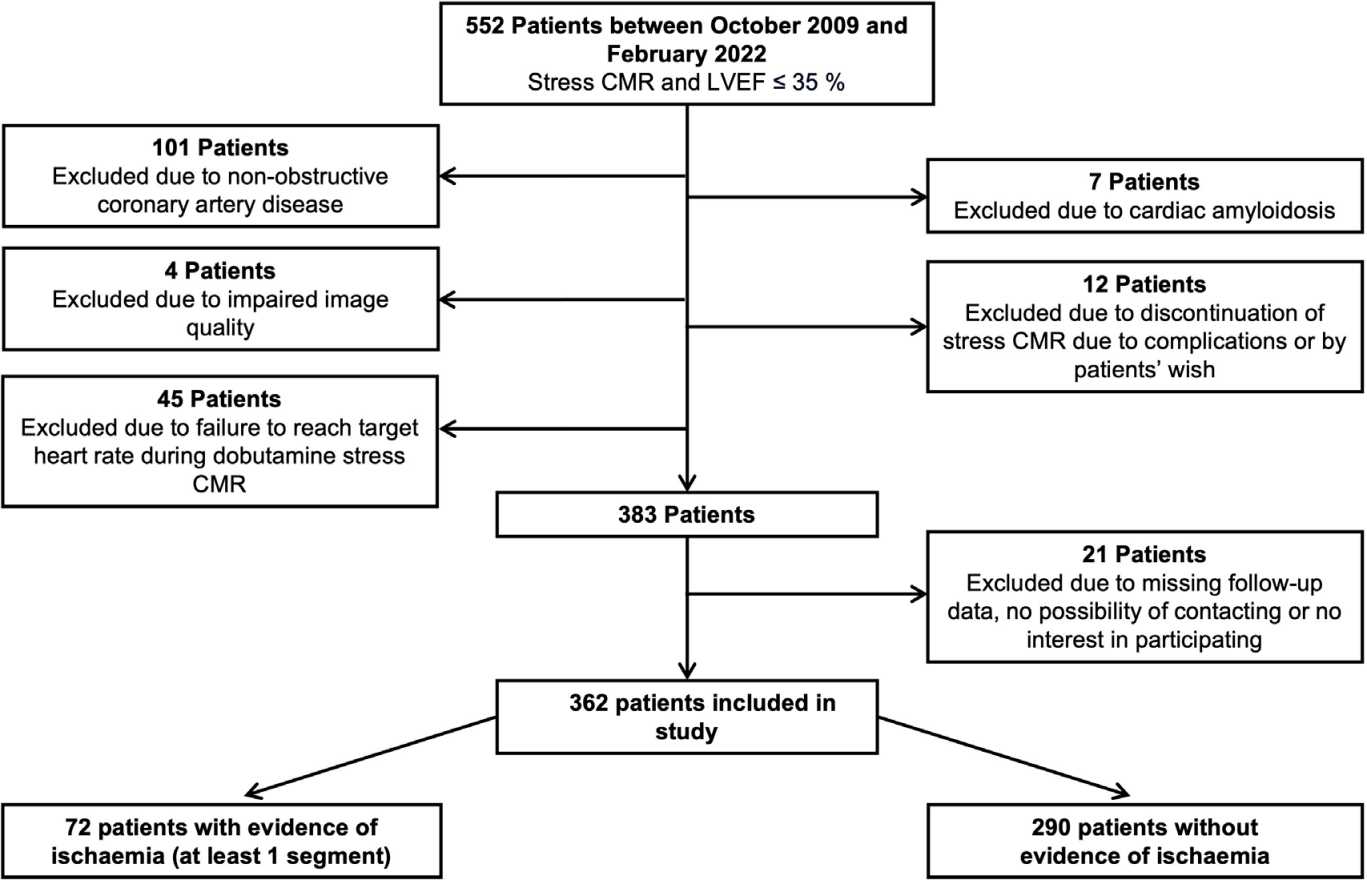
Flowchart of patient selection. Patients who were contacted for follow-up were asked for consent for participation. CMR, cardiovascular magnetic resonance; LVEF, left ventricular ejection fraction.

Of the 552 patients referred for stress CMR with documented LVEF ≤35%, 362 patients fulfilled the inclusion criteria and comprised the final study cohort (figure 1). 85.6% of the study population were men, with a median age of 70.5 (63.0–78.0) years (table 1). The medical history included an MI in 207 patients (57.2%) and a CABG in 83 patients (22.9%). 245 patients (67.7%) had three vessel CAD. 67 patients (18.5%) had left main CAD (table 2).

**Table 1:** Clinical characteristics, CMR results and laboratory results for the study population and stratified by presence of ischaemia

Patient characteristics	All (n=362)	Ischaemia (n=72)	No ischaemia (n=290)	P value
Age	70.5 (63.0–78.0)	73.9 (64.0–78.5)	70.0 (63.0–77.0)	0.3
Male, n (%)	309 (85.6)	57 (79.1)	266 (91.7)	0.1
BMI (kg/m^2^)	26.9 (24.1–29.4)	26.8 (23.7–28.8)	26.9 (24.2–29.6)	0.6
Cardiovascular RF				
Hypertension, n (%)	310 (85.6)	59 (81.9)	251 (86.5)	0.3
Hypercholesterinaemia, n (%)	272 (75.1)	52 (72.2)	220 (75.8)	0.5
Smoking, n (%)	175 (48.3)	28 (38.8)	147 (50.6)	0.1
Diabetes, n (%)	131 (36.2)	27 (37.5)	104 (35.8)	0.7
Positive family history, n (%)	107 (29.6)	22 (30.5)	85 (29.3)	0.8
Atrial fibrillation, n (%)	105 (30.9)	23 (31.9)	82 (28.3)	0.7
Perfusion stress CMR, n (%)	94 (26.2)	19 (26.3)	75 (25.8)	0.9
Dobutamine stress CMR, n (%)	268 (73.8)	53 (73.6)	215 (74.1)	0.9
LVEF, %	30.2 (25.2–33.0)	29.9 (25.3–33.5)	30.2 (25.2–32.9)	0.7
LVEDV/BSA, mL/m^2^	119.1 (101.5–144.3)	119.5 (97.8–148.7)	118.9 (101.7–141.6)	0.7
LVESV/BSA, mL/m^2^	83.1 (70.8–103.8)	85.7 (69.5–108.1)	82.8 (70.7–103.7)	0.8
LV mass, g	136.0 (115.0–166.0)	143.0 (121.0–178.0)	134.0 (115.0–161.5)	0.04
LGE imaging performed, n (%)	301 (83.1)	59 (81.9)	242 (83.4)	0.7
Infarct-like LGE, n (%)	262 (87.0)	53 (89.8)	209 (86.4)	0.4
Infarct-like LGE segments, number (IQR)	7 (4–9)	6 (3–9)	7 (4–9)	0.2
NT-proBNP, ng/L (n=139)	3516.0 (1262.5–8214.3)	3993.5 (1820.0–9261.0)	2797.0 (1191.0–7878.8)	0.3
hs-TnT, pg/mL (n=158)	42.5 (24.5–90.0)	34.0 (21.8–96.0)	43.0 (24.6–89.0)	0.4
GFR, mL/min/1.73 m^2^ (n=163)	70.8 (55.8–83.6)	70.8 (54.3–87.9)	70.8 (56.0–82.4)	0.9

Values are median (IQR) or n (%). Differences between groups were calculated using t-test, Mann-Whitney U test or χ2 test. GFR was calculated with Cockcroft and Gault formula.

BMI, body mass index; BSA, body surface area; CMR, cardiovascular magnetic resonance; GFR, glomerular filtration rate; hs-TnT, high sensitive troponin T; LGE, late gadolinium enhancement; LV, left ventricular; LVEDV, left ventricular end-diastolic volume; LVEF, left ventricular ejection fraction; LVESV, left ventricular end-systolic volume; NT-proBNP, N-terminal pro-B-type natriuretic peptide; RF, risk factor.

**Table 2:** Coronary artery disease in the study population

	All (n=362)
Left main, n (%)	67 (18.5)
Single vessel disease, n (%)	20 (5.5)
Two vessel disease, n (%)	97 (26.8)
Three vessel disease, n (%)	245 (67.7)
CTO, n (%)	132 (36.5)
History of CABG, n (%)	83 (22.9)
History of MI, n (%)	206 (57.2)
ICA before CMR, n (%)	301
Time difference ICA-CMR, days	58 (3–285)
ICA after CMR, n (%)	61
Time difference ICA-CMR, days	9 (2–58)

Values are median (IQR) or n (%).

CABG, coronary artery bypass grafting; CMR, cardiovascular magnetic resonance; CTO, chronic total occlusion; ICA, invasive coronary angiography; MI, myocardial infarction.

### CMR imaging results

A total of 288 patients (74.0%) underwent dobutamine stress CMR. Of those patients, 133 (31.2%) had peak stress perfusion in addition to wall motion analysis. 93 patients (25.7%) underwent adenosine stress CMR, and one patient (0.3%) underwent regadenoson stress CMR. The high percentage of patients undergoing dobutamine stress CMR was due to the composition of the study cohort. Nearly one quarter of the patients had CABG, and 36.5% had angiography-proven chronic total occlusion. Additionally, in patients who underwent LGE imaging, a median of seven segments exhibited infarct-like LGE patterns, indicating substantial myocardial scarring. 247 patients (68.2%) had at least one segment with probably non-viable myocardium (≥50% transmurality on LGE images). The median LVEF was 30.2 (25.2–33.0)%.

Ischaemia was evident in 72 patients (19.9%) in 3 (2-4) segments: Of those, 19 patients underwent adenosine stress CMR with 2 (2-4) ischaemic segments and 23 underwent dobutamine stress CMR showing 2 (1–4) ischaemic segments. In 30 patients positive for ischaemia, dobutamine stress CMR combined with peak perfusion imaging was performed, showing 3 (2–4) ischaemic segments (3 (2–4) segments WMAs in 14 patients, 2 (1–3) segments perfusion abnormalities in 25 patients).

### Early revascularisation

Among 72 patients with ischaemia, only 32 patients (8.8%) received early revascularisation. 40 patients (11.0%) did not receive revascularisation despite positive stress CMR. 24 of these 40 patients had only mild ischaemia: 15 patients had only one segment of ischaemia, and 9 patients had two segments of ischaemia.

Despite no ischaemia on stress CMR, 4 patients (1.1%) underwent early revascularisation with CABG due to severe three-vessel disease and 16 patients (4.4%) underwent early revascularisation with PCI due to persistent symptoms.

### Clinical endpoints

Median follow-up was 4.5 (3.0–6.6) years. 101 patients (27.9%) reached the primary endpoint: 34 patients (9.4%) suffered a cardiac death and 47 patients (13.0%) suffered a non-fatal MI. 9 patients (2.5%) survived cardiac death, 11 patients (3.0%) required ICD-shock for ventricular fibrillation.

116 patients (32.0%) reached the secondary endpoint: 32 patients (8.8%) were hospitalised for acute decompensated HF, 56 patients (15.5%) underwent PCI and 14 patients (3.7%) required CABG. 14 patients (5.0%) had an arrhythmic event, defined as ICD-terminated VT (4.1%) or external electrical cardioversion for VT without loss of spontaneous circulation (0.8%).

There was no significant difference in primary and secondary endpoints between patients with ischaemia and those without (table 3). The only outcome that differed was late revascularisation with CABG, which was more frequent in patients with ischaemia. There were no significant differences in outcomes between patients with early revascularisation and those without (online supplemental table 1).

**Table 3:** Clinical endpoints reached during follow-up

Clinical endpoint, n (%)	All (n=362)	Ischaemia (n=72)	No ischaemia (n=290)	P value
MACE	101 (27.9)	21 (29.2)	80 (27.6)	0.8
Cardiac death	41 (11.3)	10 (13.9)	31 (10.7)	0.4
Non-fatal MI	40 (11.0)	6 (8.3)	34 (11.7)	0.4
STEMI	2 (0.5)	0	2 (0.7)	0.5
Non-STEMI	38 (10.5)	6 (8.3)	32 (11.0)	0.5
Survived SCD	9 (2.5)	3 (4.2)	6 (2.1)	0.3
ICD shock for VF	11 (3.0)	2 (2.8)	9 (3.1)	0.9
Secondary endpoint	117 (32.3)	21 (29.2)	96 (33.1)	0.5
Heart failure hospitalisation	33 (9.1)	4 (5.6)	29 (10.0)	0.2
PCI >90 days after CMR	56 (15.5)	7 (9.7)	49 (16.9)	0.1
CABG >90 days after CMR	14 (3.7)	6 (8.3)	8 (2.8)	0.04
Arrhythmia	14 (5.0)	4 (5.6)	10 (3.4)	0.4
ICD shock for VT	12 (4.1)	3 (5.5)	9 (3.8)	0.5
VT	2 (0.8)	1 (1.4)	1 (0.7)	0.6
Non-cardiac death	90 (24.9)	19 (26.3)	71 (24.4)	0.7
All-cause death	144 (39.8)	32 (44.4)	112 (38.6)	0.3
Early revascularisation (CABG/PCI)	52 (14.4)	32 (44.4)	20 (6.9)	<0.0001
PCI	39 (10.8)	23 (31.9)	16 (5.5)	<0.0001
CABG	13 (3.6)	9 (12.5)	4 (1.4)	<0.0001
ICD implantation	141 (39.0)	24 (33.3)	117 (40.3)	0.8

CABG, coronary artery bypass graft; CMR, cardiovascular magnetic resonance; ICD, implantable cardioverter defibrillator; MACE, major adverse cardiovascular event; MI, myocardial infarction; PCI, percutaneous coronary intervention; SCD, sudden cardiac death; STEMI, ST-elevation myocardial infarction; VF, ventricular fibrillation; VT, ventricular tachycardia.

### Survival analysis and prognostic value

In univariate regression analyses, ischaemia on stress CMR, irrespective of whether revascularisation was performed, was not significantly associated with MACE overall (HR 1.20, 95% CI 0.74 to 1.95, p=0.4, online supplemental file 1). Similarly, when ischaemic burden was analysed (‘number of ischaemic segments’), it was not significantly associated with MACE. Similarly, ischaemia was not significantly associated with the secondary endpoint in univariate analysis, neither was ischaemic burden.

**Figure 2:**
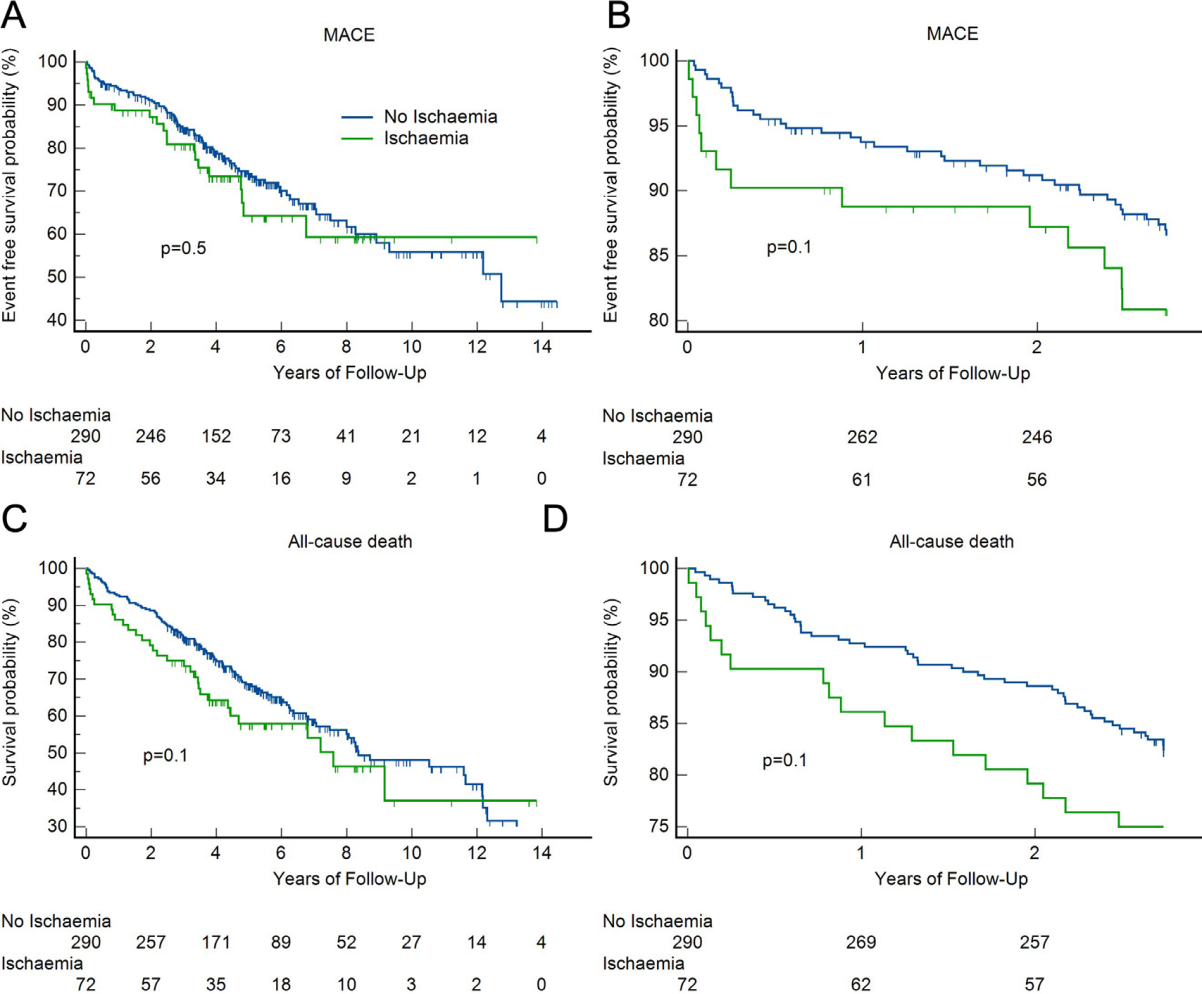
Survival analysis for MACE and all-cause death stratified by presence of ischaemia on stress CMR after complete median follow-up of 4.5 years (MACE: A, all-cause death: C) and in interim analysis after 1000 days (MACE: B, all-cause death: D). CMR, cardiovascular magnetic resonance; MACE, major adverse cardiovascular event.

Kaplan-Meier cumulative event rate for MACE stratified by the presence versus absence of inducible ischaemia revealed no significant difference between the two groups (figure 2A). In an interim analysis after 1000 days, the separation of the two curves in the first 3 years was evident, but not significant (figure 2B).

In a subsequent analysis, patients with early revascularisation due to ischaemia were reassigned to the no ischaemia group. In this analysis, ischaemia was not significantly associated with MACE (HR 1.32, 95% CI 0.74 to 2.38, p=0.4) or the secondary endpoint (HR 1.08, 95% CI 0.59 to 1.97, p=0.8).

Additionally, all patients undergoing early revascularisation were censored, resulting in a group of 310 patients (270 patients without ischaemia (87.1%) and 40 patients with ischaemia (12.9%)). In this analysis, we found no significant association between ischaemia and MACE (HR 1.38, 95% CI 0.76 to 2.49, p=0.3) or the secondary endpoint (HR 1.14, 95% CI 0.62 to 2.08, p=0.7). Regression analysis showed no significant difference between patients with and without ischaemia (MACE p=0.3, secondary endpoint p=0.7). There was no significant difference in event-free survival probability for the secondary endpoint between patients with and those without ischaemia (online supplemental central illustration).

Ischaemia on stress CMR, whether evaluated as dichotomous or a discrete variable, was not significantly associated with all-cause death. The Kaplan-Meier curve showed no significant difference in all-cause death when stratifying for ischaemia, even though the curves separate early on (figure 2C). An interim analysis after 1000 days similarly showed no significant difference between the two groups (figure 2D).

Early revascularisation was not significantly associated with MACE or the secondary combined endpoint in survival analysis, but showed significant association with all-cause death (HR 1.55, 95% CI 1.01 to 2.37, p=0.04). After stratifying patients by early revascularisation in the Kaplan-Meier curve, there was a significant difference in all-cause death, with a worse outcome for patients who received early revascularisation (online supplemental figure 1). There was no significant difference when stratifying for MACE or the combined secondary endpoint (p=0.5 and p=0.4, respectively).

LVEF was significantly associated with MACE (HR 0.95, 95% CI 0.91 to 0.98, p=0.002) and all-cause death (HR 0.95, 95% CI 0.93 to 0.98, p=0.003). Indexed left ventricular end-diastolic volume (LVEDV) and left ventricular end-systolic volume (LVESV) were significantly associated with MACE (LVEDV/body surface area (BSA) HR 1.01, 95% CI 1.00 to 1.02, p=0.0003; LVESV/BSA HR 1.01, 95% CI 1.00 to 1.02, p=0.0001), the combined secondary endpoint and all-cause death (online supplemental tables 2 and 3). LV mass was associated with the combined secondary endpoint and all-cause death (HR 1.01, 95% CI 1.00 to 1.01, p=0.006 and HR 1.01, 95% CI 1.00 to 1.01, p=0.009, respectively). Neither the presence of infarct-like LGE nor the number of myocardial segments with infarct-like LGE was associated with MACE, the combined secondary endpoint or all-cause death (online supplemental tables 2 and 3).

### Complications during stress CMR

While complications occurred more frequently during dobutamine CMR than during perfusion stress CMR, the difference was not statistically significant (online supplemental table 4). One patient (0.3%) experienced sustained VT, which was successfully terminated with beta blocker administration. Another patient (0.3%) had multiple episodes of non-sustained VT that were resolved with beta blocker therapy. Atrial fibrillation occurred in two patients (0.6%) during the stress test: In one case, it resolved with beta blocker administration; the other required electrical cardioversion. Frequent premature ventricular contractions were observed in four patients (1.2%) undergoing dobutamine stress testing and in one patient (0.3%) during adenosine stress testing.

## Discussion

The aim of this study was to evaluate the prognostic value of ischaemia assessed by stress CMR in patients with IHD and LVEF ≤35%. Ischaemia was not a significant predictor of the primary endpoint of MACE, the combined secondary endpoint, or all-cause mortality.

### Comparison with previous studies

To the best of our knowledge, this is the first study to specifically examine the prognostic value of stress CMR in patients with IHD and severely reduced LVEF. In a study investigating the prognostic value of dobutamine stress CMR in 200 patients with LVEF ≤55%, ischaemia was not associated with cardiovascular events in the subgroup of patients with LVEF <40%.^18^ Similar results were found in another trial investigating the prognostic value of dobutamine stress CMR in a cohort of 1493 all-comers, showing no significant prognostic value of ischaemia in the subgroup of patients with LVEF ≤35%.^19^ Of note, both studies also included patients with non-ischaemic dilatative cardiomyopathy. It is of particular interest that the Kaplan-Meier curves for MACE and all-cause death initially diverge, indicating worse survival for patients with ischaemia in the first few years, although this difference was not statistically significant. After about 7 years, the curves cross and eventually show no significant difference, suggesting that the differences between the groups disappear over time. One may hypothesise that in this vulnerable cohort, patients without ischaemia in the initial exam develop ischaemia later on, leading to convergence of the curves over time. Current literature portends a warranty period of approximately 3.5 years for a negative stress CMR.^20^ Additionally, in this cohort with a high burden of disease, HF and its associated complications and comorbidities may have more impact on long-term survival and confound group differences.

### Survival and revascularisation

Recent data has reignited the debate over the prognostic role of ischaemia and consecutive revascularisation in patients with CAD. The ISCHEMIA trial included 5179 subjects and showed no improvement of cardiovascular outcome for early revascularisation in patients with ischaemia.^3^ Similarly, the REVIVED-BCIS trial recently showed that patients with severely reduced LVEF due to extensive CAD did not benefit from the addition of revascularisation to optimal medical therapy.^2^ And while the extent of ischaemia improved after revascularisation in the sham-controlled ORBITA trial, an improvement of exercise capacity could not be demonstrated.^21^

In our study, there was no significant difference in MACE between patients who received revascularisation and those who did not. Interestingly, only about half of patients with ischaemia received early revascularisation, either by PCI or CABG. Patients who did not receive early revascularisation despite ischaemia mainly had a mild ischaemic burden (1–2 segments). Previous studies have identified an ischaemic burden of 1.5 segments associated with cardiovascular endpoints in patients without reduced LVEF, and current guidelines of the European Society of Cardiology recommend revascularisation when at least 10% of LV myocardium shows ischaemia.^22 23^ This may have influenced the decision to forgo revascularisation.

The current study found a higher all-cause mortality in patients who underwent early revascularisation, regardless of ischaemia. This result must be taken with caution, because the majority of deaths were attributed to non-cardiac causes. In this vulnerable population, early coronary revascularisation may have been pursued not necessarily because of objective ischaemia alone, but because it was seen as a last therapeutic option for patients with advanced disease.

### Dobutamine and perfusion imaging

A recurring question is the accuracy of stress testing in patients with severely reduced LVEF. Particularly in patients with extensive CAD and multivessel disease, which may cause balanced ischaemia, adenosine perfusion imaging may carry a risk of false-negative results.^24^ In addition, the haemodynamic response to adenosine appears to be blunted in patients with severely reduced LVEF, more often requiring an increase in adenosine dose up to 210 µg/kg/min to achieve an adequate haemodynamic response.^25^

Therefore, in our centre, dobutamine is chosen as a stress agent in cases where the accuracy of adenosine is expected to be impaired due to balanced ischaemia, such as in the case of CABG and chronic total occlusion. Based on previous data showing improved accuracy in identifying patients at increased risk of cardiovascular events, wall motion analysis is complemented by perfusion imaging during peak dobutamine stress.^19^ The diagnostic value of dobutamine stress CMR has been extensively demonstrated,^26 27^ and the prognostic accuracy of adenosine and dobutamine stress CMR has been shown to be equivalent.^28^

### Clinical implications

The results indicate that ischaemia may not be the most important determinant in IHD with severely reduced LVEF. While ischaemia has been regarded as a therapeutic target in the management of IHD, our findings align with an evolving understanding that structural and functional myocardial parameters may carry greater prognostic weight, particularly in patients with advanced ventricular dysfunction. LV volumes and LVEF have previously been shown to have strong prognostic value in patients with IHD.^29^ This was confirmed in our results, where extensive LV remodelling—reflected by significantly increased end-diastolic and end-systolic volumes—was prominent and was significantly associated with adverse clinical outcomes. Ventricular remodelling is a key marker of myocardial disease progression, indicating chronic myocardial injury.^30^

Unlike previous studies, ischaemic LGE did not have significant prognostic value. One possible explanation is the high prevalence of ischaemic LGE in our study population, which may have limited its ability to serve as a discriminator. Previous studies involved patients with a lower prevalence and extent of LGE. Ge *et al* analysed 582 patients with reduced LV function; 277 of these patients (48%) had a median of 4 (range 2–7) ischaemic LGE segments.^10^ In another study, Pezel *et al* analysed patients with LVEF <40%; 57.3% of this cohort exhibited ischaemic LGE, with a mean of 3.7±2.1 segments.^11^ Additionally, the prognostic value of ischaemic LGE may have been overshadowed by other, more dominant clinical factors in this cohort, such as reduced LVEF, comorbid conditions or advanced CAD stage. These factors could have played a more decisive role in determining outcomes, thus diminishing the independent predictive value of LGE.

Our results point to the need for a comprehensive approach in patients with IHD and severely reduced LVEF that includes factors other than pure ischaemic burden, such as patient characteristics, volumetric measurements and shared decision making.

### Limitations

First, stress perfusion was analysed visually and not quantitatively. However, the studies were analysed by two experienced readers, each with at least 5 years of experience in CMR. Second, although follow-up data were available for the majority of subjects, the cause of death could not always be definitively determined and, in cases of uncertainty, was classified as non-cardiac death. Third, similar to previous studies, the majority of our study cohort was men, making clinical conclusions for women difficult. Finally, the number of patients with ischaemia who received revascularisation is relatively small and we cannot exclude an underlying positive effect of revascularisation on this small subgroup.

## Conclusion

In a large cohort of patients with IHD and severely reduced LVEF, the presence of ischaemia derived from stress CMR was not associated with cardiovascular prognosis or all-cause death.

## Supplementary material

10.1136/openhrt-2025-003466 online supplemental file 1

10.1136/openhrt-2025-003466 online supplemental file 2

## Data Availability

Data are available upon reasonable request.
