# Supplementary material for: Prognostic value of stress cardiovascular magnetic resonance in patients with ischaemic heart disease and severely reduced left ventricular ejection fraction
Source: Open Heart. 2025 Aug 26;12(2):e003466. doi: 10.1136/openhrt-2025-003466 (PMC12382561; doi:10.1136/openhrt-2025-003466)
Supplement: online supplemental file 1 [file openhrt-12-2-s001.docx]

**Prognostic value of stress cardiovascular magnetic resonance in patients with ischemic heart disease and severely reduced left ventricular ejection fraction**

Ailís Ceara Haney, Janek Salatzki, Andreas Ochs, Thomas Hilbel, Lukas D. Weberling, Hauke Hund, Evangelos Giannitsis, Norbert Frey, Henning Steen, Dirk Loßnitzer, Florian André

**Supplementary Material**

**Methods**

**CMR imaging analysis**

Cine long axis 2-, 3-, and 4-chamber views as well as a cine short-axis stack covering the entire LV were obtained using a breath-hold steady-state free precession sequence (bSSFP) with a slice thickness of 8 mm. 40 (Achieva) or 35 (Ingenia CX and Ingenia) phases per cardiac cycles were acquired. CMR series were analyzed using a dedicated software (cvi42 Version 5.6.6, Circle Cardiovascular Imaging, Calgary, Canada). LV end-diastolic (EDV) and end-systolic (ESV) volumes, LVEF, and LV myocardial mass (LV mass) were obtained in short axis stacks by manually tracing epi- and endocardial borders, using a smooth approach adding papillary muscles and trabeculae to the LV cavity. Late gadolinium enhancement (LGE) images were acquired 10 min after administration of gadopentetic acid (Magnograf, Schering, Berlin, Germany) (before February 2016) or Gadobutrol (Gadovist, Bayer Vital, Leverkusen, Germany) (after January 2016). The number of ischemic LGE positive segments was assessed according to the 17-segment model of the American Heart Association (AHA) (14). Ischemic LGE was defined as subendocardial or transmural LGE pattern. Non-viable myocardium was defined by presence of ≥50% LGE, as previously published (13).

**Vasodilator stress CMR**

Stress perfusion imaging was performed using a continuous intravenous infusion of adenosine for at least three minutes at a rate of 140 μg/kg body weight/min or 210 μg/kg body weight/min, in case of an inadequate hemodynamic response.

In patients with severe obstructive lung disease (e.g., asthma), regadenoson stress CMR was performed. Regadenoson stress perfusion images were acquired approximately 60 seconds after the administration of 0.4 mg regadenoson i.v. (Rapiscan; GE Healthcare, Chicago, United States of America).

Myocardial perfusion imaging was conducted in three LV short-axis slices (apical, mid-ventricular, and basal). An inducible ischaemia was visually deemed present in case of hypoperfusion of at least 1 AHA segment during perfusion, not evident at baseline, which was accompanied by a perfusion/LGE mismatch in the presence of late gadolinium enhancement (LGE). In each patient, the total number of ischemic segments was assessed using the 16-segment model of the AHA (14).

**Dobutamine stress CMR**

Dobutamine stress CMR was performed as recommended by the Society for Cardiovascular Magnetic Resonance (15). Cine 2-, 3- and 4-chamber views and three short-axis views (apical, mid-ventricular, and basal) were acquired. Dobutamine was infused during 3-minute stages at incremental doses of 10, 20, 30, and 40 μg/kg of body weight/min until at least 85% of the age-predicted heart rate was reached (220-age in years). Atropine was administered in 0.25 mg increments (up to a maximal dose of 2.0 mg) if the target heart rate was not reached. Images were assessed for wall motion abnormalities at rest and at low, intermediate, and maximum stress. During stress CMR, symptoms, heart rate, peripheral blood pressure, and oxygen saturation were continuously monitored. Stress testing was stopped when target heart rate was reached or when new or worsening wall motion abnormalities (WMA) in at least 1 segment were documented.

**Patient follow-up and outcome**

Cardiac death was defined as death preceded by acute MI, acute HF, or documented fatal arrhythmias and survived sudden cardiac death was defined as return of spontaneous circulation with cardiopulmonary resuscitation due to a cardiopulmonary cause. Non-fatal MI was defined according to the fourth universal definition of acute MI (18). Hospitalization for HF was defined as symptomatic HF requiring hospitalization, evidence of pulmonary edema on chest X-ray, and/or impaired systolic LV function.

PCI or CABG in the first 90 days after CMR were censored, to exclude bias for patients referred for revascularization after index CMR. In cases where several events occurred, only the first event was considered for event-free survival analysis. In cases where patients survived sudden cardiac death or received adequate ICD-therapy due to ventricular fibrillation, with diagnosis of MI the same day, reanimation/adequate ICD-therapy was counted to event-free survival analysis. The combined primary and secondary endpoint were counted and analyzed separately. **Supplementary Figure**


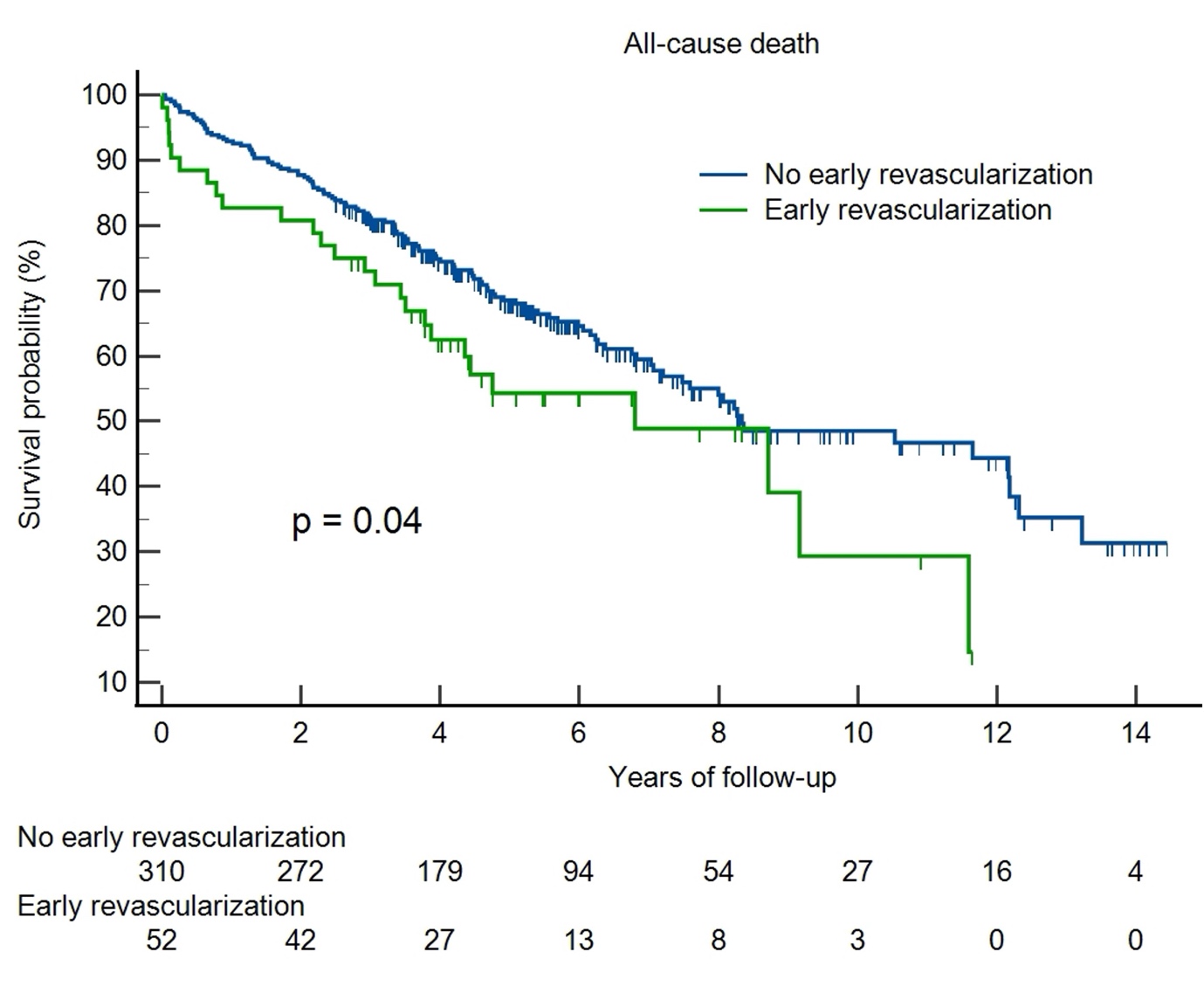


**Supplementary Figure 1:** Survival analysis for all-cause death stratified by early revascularisation after stress CMR (irrespective of presence of ischaemia), showing worse survival for patients who received early revascularisation.

**Supplementary Tables**

**Supplementary Table 1:** Clinical endpoints reached during follow-up, differentiated by early revascularization.

ACS – acute coronary syndrome. CABG – coronary artery bypass graft. ICD – implantable cardioverter defibrillator. MACE – major adverse cardiovascular events. MI – myocardial infarction. Non-STEMI – non-ST-elevation myocardial infarction. PCI – percutaneous coronary intervention. SCD – sudden cardiac death. STEMI – ST-elevation myocardial infarction. VT – ventricular tachycardia.

| **Clinical endpoint, n (%)** | **All (n = 362)** | **Early Revascularization (n = 52)** | **No revascularization (n = 310)** | **p-value** |
| --- | --- | --- | --- | --- |
| **MACE** | 101 (27.9) | 15 (28.8) | 86 (27.7) | 0.8 |
| Cardiac death | 41 (11.3) | 6 (11.5) | 35 (11.3) | 0.9 |
| Non-fatal MI | 40 (11.0) | 5 (9.6) | 35 (11.3) | 0.7 |
| STEMI | 2 (0.5) | 0 | 2 (0.7) | 0.5 |
| Non-STEMI | 38 (10.5) | 5 (9.6) | 33 (10.6) | 0.8 |
| Survived SCD | 9 (2.5) | 2 (3.8) | 7 (2.3) | 0.5 |
| ICD shock for VF | 11 (3.0) | 2 (2.9) | 9 (3.8) | 0.7 |
| **Secondary Endpoint** | 116 (32.0) | 18 (34.6) | 99 (31.9) | 0.7 |
| Heart Failure Hospitalization | 33 (8.8) | 4 (7.7) | 29 (9.4) | 0.7 |
| PCI > 90 days after CMR | 56 (15.5) | 9 (17.3) | 47 (15.2) | 0.7 |
| CABG > 90 days after CMR | 14 (3.7) | 4 (7.7) | 10 (3.2) | 0.1 |
| Arrhythmia | 14 (5.0) | 1 (1.9) | 13 (4.2) | 0.4 |
| ICD shock for VT | 12 (4.1) | 1 (1.9) | 11 (3.5) | 0.5 |
| VT | 2 (0.8) | 0 | 2 (0.6) | 0.5 |
| Non-cardiac death | 90 (24.9) | 17 (32.7) | 73 (23.5) | 0.2 |
| All-cause death | 144 (39.8) | 26 (50.0) | 120 (38.7) | 0.1 |
| ICD implantation | 141 (39.0) | 17 (32.7) | 124 (40.0) | 0.3 |

**Supplementary Table 2:** Univariate Cox regression analysis for major adverse cardiovascular events and the secondary combined endpoint.

BMI – body mass index. BSA – body surface area. LGE – late gadolinium enhancement. LVEDV – left ventricular end-diastolic volume. LVEF – left ventricular ejection fraction. LVESV – left ventricular end-systolic volume. MI – myocardial infarction.

| Parameter | P-value | HR (95 % CI) | C-index (95 % CI) |
| --- | --- | --- | --- |
| Major adverse cardiovascular events | | | |
| Age | 0.1 | 1.02 (0.99 – 1.04) | 0.54 (0.48 – 0.60) |
| Sex | 0.7 | 1.09 (0.63 – 1.90) | 0.52 (0.47 – 0.56) |
| BMI | 0.2 | 0.97 (0.93 – 1.01) | 0.58 (0.51 – 0.64) |
| Diabetes | 0.04 | 1.51 (1.01 – 2.25) | 0.56 (0.51 – 0.61) |
| Hypertension | 0.3 | 1.42 (0.76 – 2.65) | 0.51 (0.47 – 0.55) |
| Smoking | 0.7 | 0.93 (0.63 – 1.38) | 0.49 (0.44 – 0.54) |
| Dyslipidemia | 0.9 | 0.97 (0.62 – 1.51) | 0.51 (0.47 – 0.56) |
| History of MI | 0.7 | 0.92 (0.62 – 1.37) | 0.51 (0.46 – 0.57) |
| LVEF | 0.002 | 0.95 (0.91 – 0.98) | 0.59 (0.54 – 0.65) |
| LVEDV/BSA | 0.0003 | 1.01 (1.00 – 1.02) | 0.59 (0.53 – 0.64) |
| LVESV/BSA | 0.0001 | 1.01 (1.00 – 1.02) | 0.61 (0.55 – 0.67) |
| LV mass | 0.5 | 1.00 (0.99 – 1.01) | 0.51 (0.44 – 0.57) |
| LGE, dichotomous | 0.3 | 1.55 (0.71 – 3.39) | 0.52 (0.48 – 0.55) |
| LGE, discrete | 0.5 | 1.02 (0.95 – 1.10) | 0.52 (0.46 – 0.59) |
| Ischaemia, dichotomous | 0.4 | 1.20 (0.74 – 1.95) | 0.52 (0.47 – 0.57) |
| Ischaemia, discrete | 0.7 | 0.97 (0.83 – 1.13) | 0.48 (0.43 – 0.52) |
| Early revascularisation | 0.5 | 1.19 (0.67 – 2.06) | 0.52 (0.48 – 0.60) |
| Secondary Combined Endpoint | | | |
| Age | 0.8 | 1.00 (0.98 – 1.02) | 0.50 (0.44 – 0.56) |
| Sex | 0.6 | 0.84 (0.48 – 1.48) | 0.51 (0.48 – 0.54) |
| BMI | 0.5 | 1.00 (0.99 – 1.01) | 0.50 (0.44 – 0.55) |
| Diabetes | 0.1 | 1.40 (0.97 – 2.03) | 0.53 (0.49 – 0.58) |
| Hypertension | 0.2 | 1.43 (0.80 – 2.54) | 0.53 (0.51 – 0.56) |
| Smoking | 0.3 | 1.20 (0.83 – 1.73) | 0.53 (0.48 – 0.58) |
| Dyslipidemia | 0.01 | 1.82 (1.13 – 2.92) | 0.56 (0.52 – 0.57) |
| History of MI | 0.2 | 1.28 (0.87 – 1.87) | 0.53 (0.49 – 0.58) |
| LVEF | 0.1 | 0.97 (0.94 – 1.01) | 0.53 (0.47 – 0.59) |
| LVEDV/BSA | <0.0001 | 1.01 (1.00 – 1.02) | 0.59 (0.53 – 0.65) |
| LVESV/BSA | <0.0001 | 1.01 (1.01 – 1.02) | 0.59 (0.53 – 0.64) |
| LV mass | 0.006 | 1.01 (1.00 – 1.01) | 0.56 (0.51 – 0.62) |
| LGE, dichotomous | 0.4 | 1.31 (0.68 – 2.53) | 0.54 (0.51 – 0.56) |
| LGE, discrete | 0.3 | 0.97 (0.91 – 1.03) | 0.54 (0.48 – 0.60) |
| Ischaemia, dichotomous | 0.9 | 0.99 (0.61 – 1.60) | 0.49 (0.45 – 0.53) |
| Ischaemia, discrete | 0.4 | 1.06 (0.93 – 1.20) | 0.52 (0.47 – 0.56) |
| Early revascularisation | 0.5 | 1.18 (0.71 – 1.98) | 0.52 (0.48 – 0.56) |

**Supplementary Table 3:** Univariate Cox regression analysis for all-cause death.

BMI – body mass index. BSA – body surface area. LGE – late gadolinium enhancement. LVEDV – left ventricular end-diastolic volume. LVEF – left ventricular ejection fraction. LVESV – left ventricular end-systolic volume. MI – myocardial infarction.

| CMR parameter | P-value | HR (95 % CI) | C-index (95 % CI) |
| --- | --- | --- | --- |
| All-cause death | | | |
| Age | <0.0001 | 1.05 (1.03 – 1.07) | 0.62 (0.57 – 0.67) |
| Sex | 0.4 | 1.23 (0.79 – 1.92) | 0.52 (0.49 – 0.55) |
| BMI | 0.5 | 0.99 (0.98 – 1.01) | 0.54 (0.49 – 0.60) |
| Diabetes | 0.0002 | 1.86 (1.34 – 2.58) | 0.58 (0.54 – 0.63) |
| Hypertension | 0.1 | 1.56 (0.90 – 2.71) | 0.52 (0.48 – 0.55) |
| Smoking | 0.2 | 0.81 (0.59 – 1.13) | 0.52 (0.48 – 0.57) |
| Dyslipidemia | 0.5 | 1.15 (0.78 – 1.69) | 0.50 (0.46 – 0.54) |
| History of MI | 0.7 | 0.92 (0.61 – 1.40) | 0.51 (0.46 – 0.57) |
| LVEF | 0.003 | 0.95 (0.93 – 0.98) | 0.58 (0.53 – 0.63) |
| LVEDV/BSA | 0.01 | 1.01 (1.00 – 1.01) | 0.56 (0.51 – 0.62) |
| LVESV/BSA | 0.009 | 1.01 (1.00 – 1.02) | 0.59 (0.52 – 0.65) |
| LV mass | 0.009 | 1.01 (1.00 – 1.01) | 0.54 (0.49 – 0.59) |
| LGE, dichotomous | 0.9 | 1.02 (0.58 – 1.78) | 0.50 (0.47 – 0.54) |
| LGE, discrete | 0.3 | 0.97 (0.91 – 1.03) | 0.51 (0.44 – 0.57) |
| Ischaemia, dichotomous | 0.1 | 1.34 (0.91 – 1.99) | 0.54 (0.50 – 0.57) |
| Ischaemia, discrete | 0.3 | 1.06 (0.95 – 1.18) | 0.54 (0.50 – 0.57) |
| Early revascularisation | 0.04 | 1.55 (1.01 – 2.37) | 0.53 (0.50 – 0.57) |

**Supplementary Table 4:** Complications during stress cardiovascular magnetic resonance.

CMR – cardiovascular magnetic resonance. PVC – premature ventricular contraction. VT – ventricular tachycardia.

| **Complication, n (%)** | All  (n = 362) | Dobutamine CMR  (n = 268) | Perfusion CMR  (n = 94) | p-value |
| --- | --- | --- | --- | --- |
| Dizziness | 1 (0.3) | 1 (0.4) | 0 | 0.5 |
| Nausea | 1 (0.3) | 0 | 1 (1.1) | 0.09 |
| Arrhythmia | 9 (2.5) | 8 (3.0) | 1 (1.1) | 0.3 |
| Atrial fibrillation | 2 (0.6) | 2 (0.7) | 0 | 0.4 |
| Frequent PVCs | 5 (1.4) | 4 (1.5) | 1 (1.1) | 0.7 |
| Non-sustained VT | 1 (0.3) | 1 (0.4) | 0 | 0.5 |
| Sustained VT | 1 (0.3) | 1 (0.4) | 0 | 0.5 |
