# Supplementary figures and images for: Prognostic value of stress cardiovascular magnetic resonance in patients with ischaemic heart disease and severely reduced left ventricular ejection fraction
Source: Open Heart. 2025 Aug 26;12(2):e003466. doi: 10.1136/openhrt-2025-003466 (PMC12382561; doi:10.1136/openhrt-2025-003466)

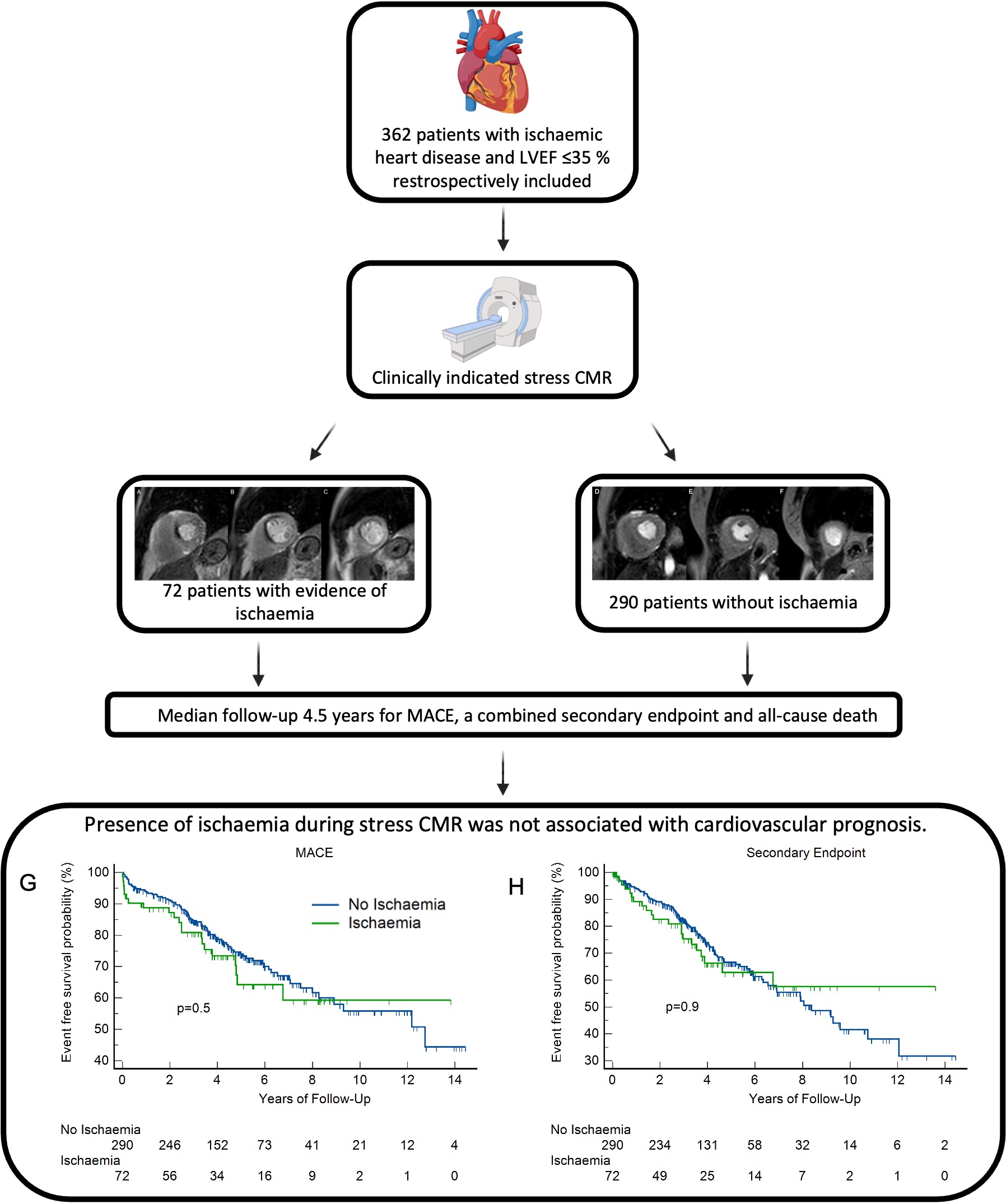

Supplement: online supplemental file 2 [file openhrt-12-2-s002.jpg]
